# Advances in Detection Methods for Human Respiratory Syncytial Virus Neutralizing Antibodies

**DOI:** 10.3390/vaccines14060550

**Published:** 2026-06-22

**Authors:** Qi Shen, Jing Gai, Yanqiu Zhou

**Affiliations:** Institute of Microbiology Laboratory, Shanghai Municipal Center for Disease Control and Prevention, Shanghai 201107, China; shenqi_2@scdc.sh.cn (Q.S.); gaijing@scdc.sh.cn (J.G.)

**Keywords:** human respiratory syncytial virus, neutralizing antibody, plaque reduction neutralization test, focus reduction neutralization test, pseudovirus, reporter virus

## Abstract

Human respiratory syncytial virus (HRSV) is a major cause of severe lower respiratory tract infections in infants, young children, and older adults worldwide. With the approval of nirsevimab and HRSV vaccines, accurate measurement of neutralizing antibody levels has become essential for vaccine evaluation, immunization strategy design, and seroepidemiology. The plaque reduction neutralization test (PRNT) remains the gold standard, but it is slow, low-throughput, and requires high biosafety. In recent years, newer methods including focus reduction neutralization testing (FRNT), pseudovirus neutralization testing (PNT), and fluorescent/luminescent reporter virus systems (RVSs) have improved speed and throughput while maintaining high specificity. This review summarizes the principles, performance, applications, and standardization challenges of these assays, offering methodological guidance for HRSV research and prevention in China.

## 1. Introduction

*Human respiratory syncytial virus (HRSV)* is an enveloped, negative-sense single-stranded RNA virus in the *Pneumovirus* genus, with two main antigenic subtypes, A and B. Globally, about 33 million children under 5 years seek care for HRSV annually, and over 3 million require hospitalization, creating a substantial disease burden [1,2]. In 2023, the long-acting monoclonal antibody nirsevimab and the maternal vaccine Abrysvo were approved, marking a new era of combined active and passive immunization [3,4]. Neutralizing antibody titers, as key correlates of protection (CoPs), now demand accurate, comparable, and accessible detection methods.

HRSV vaccines and monoclonal antibodies target the viral envelope glycoproteins: *attachment (G)* and *fusion (F)*. The F protein is the primary target of neutralizing antibodies, mediating membrane fusion and showing >90% amino acid homology between subtypes A and B [5]. F exists in *prefusion (pre-F)* and *postfusion (post-F)* conformations [6]; only pre-F displays key neutralizing epitopes, such as site Ø [7]. In 2013, McLellan et al. solved and stabilized the pre-F structure, transforming vaccine development. Stabilized pre-F antigens (e.g., DS-Cav1) strongly induce neutralizing antibodies [8]. Approved maternal vaccines (e.g., Abrysvo) and mRNA candidates use stabilized pre-F to elicit high titers of pre-F-specific neutralizing antibodies. Nirsevimab also targets pre-F site Ø, its long half-life and broad neutralization stemming from this specific epitope recognition [9,10,11]. Thus, detection methods must distinguish functional pre-F antibodies to assess vaccine immunogenicity, monitor population immunity, and guide clinical use.

An ideal assay should be specific, reproducible, moderately high-throughput, and low-risk. However, HRSV’s slow replication, atypical cytopathic effect (CPE), and syncytium formation complicate functional antibody testing. This review outlines the evolution of HRSV-neutralization assays, compares classic and emerging methods, and discusses standardization to support research and public health practice in China.

## 2. The Gold Standard for HRSV-Neutralizing Antibody Detection

### 2.1. Plaque Reduction Neutralization Test (PRNT)

PRNT reflects the functional activity of neutralizing antibodies by quantitatively determining the ability of the test serum to inhibit the formation of plaques by live viruses on a monolayer of adherent cells. The standard procedure includes the following steps: (1) serum inactivation at 56 °C for 30 min; (2) co-incubation of serially diluted serum with a fixed dose of HRSV, usually 50–100 plaque-forming units (PFUs), in a 96-well plate at 37 °C for 1–2 h; (3) inoculation of the mixture onto a confluent monolayer of HEp-2 or Vero cells (commonly in 24-well plates); (4) adsorption for 1–2 h, followed by overlay with methylcellulose or agarose semi-solid medium; and (5) incubation for 4–5 days, followed by cell fixation and staining, such as with crystal violet or neutral red, and plaque counting. The results are expressed as the serum dilution that inhibits 50% or 90% plaque formation (PRNT_50_/PRNT_90_) [12].

In vaccine research, PRNT serves as a pivotal assay for evaluating immunogenicity. For example, in a clinical trial of the live-attenuated intranasal HRSV vaccine HRSV/6120/ΔNS2/1030s in HRSV-seronegative young children, PRNT was used to quantify neutralizing antibody titers, which confirmed the strong immunogenicity and robust anamnestic neutralizing antibody responses of the vaccine following natural HRSV infection [13]. PRNT was also employed to evaluate another live-attenuated vaccine candidate, HRSV/ΔNS2/Δ1313/I1314L, verifying its genetic stability and neutralizing antibody induction specificity in the pediatric population, thereby providing critical evidence for the further development of intranasal live-attenuated HRSV vaccines [14].

#### Advantages and Limitations

PRNT directly measures the biological function of antibodies in blocking viral infections and has high specificity; thus, it has been widely adopted in international vaccine evaluation research. Quantitatively, PRNT exhibits inter-assay coefficients of variation (CV) of 12–20% [12] and intra-assay CV of 8–15%, with a diagnostic specificity of >98% and sensitivity of >95% against seropositive reference panels [15]. However, it has notable limitations; (1) it is time-consuming (5–7 days); (2) cumbersome operations relying on manual counting, leading to strong subjectivity; (3) it requires BSL-2 laboratories and a large quantity of live viruses; (4) low throughput (<100 samples/day) makes it difficult to meet the needs of large-scale serological surveys.

When PRNT fails or misleads, low-titer sera make plaque counts become sparse and variable. PRNT_50_ estimates can vary >2-fold between readers, leading to false-negative classifications in 5–10% of low-positive samples. Syncytium-rich strains lead to fused cells obscuring plaques, increasing manual counting errors to 25–35% and reducing inter-laboratory correlation (r < 0.75). Contaminated or misinactivated sera contain residual non-specific inhibitors that reduce apparent antibody titers by 2–4-fold, resulting in an artificial underestimation of neutralizing activity.

### 2.2. Progress in Novel Neutralizing Antibody Detection Methods

#### 2.2.1. Virus Reduction Neutralization Test (VRNT)

Significant improvements have been made in the speed and throughput of PRNT by adopting automated imaging technologies. A fast, high-throughput, and robust imaging-based VRNT for respiratory syncytial virus has been developed and qualified, which enables the automated quantification of infected cells using an imaging cytometer SpectraMax i3x Mini/Max cytometer in 96-well plates. This approach shortens the detection cycle to within 24 h and exhibits high robustness, reproducibility, and good correlation with conventional PRNT, making it highly suitable for large-scale serological testing and clinical vaccine trials [15]. Such an automated imaging-based platform greatly enhances the practicality and clinical applicability of neutralization assays for HRSV. Quantitatively, VRNT correlates with PRNT with Pearson r = 0.94–0.97 [15], intra-assay CV = 4–7%, inter-assay CV = 6–10%, and diagnostic AUC = 0.98 (95% CI: 0.95–1.00) relative to PRNT [15]. Such an automated imaging-based platform greatly enhances the practicality and clinical applicability of neutralization assays for HRSV.

In a comparative study of potency assays for the anti-HRSV monoclonal antibody MK-1654, three functional cell-based neutralization methods were evaluated, including imaging-based VRNT and two reporter virus-based systems (HRSV-GFP and HRSV-NLucP). All three assays exhibited acceptable dose–response relationships and comparable EC50 values for MK-1654. However, the HRSV-NLucP reporter virus assay was ultimately selected for further development and pre-qualification because of its operational simplicity and adaptability to routine quality control laboratories [16]. Nevertheless, VRNT remains a reliable high-throughput option for the initial screening and functional evaluation of neutralizing mAbs during the early stages of drug development.

When VRNT fails or misleads, several practical limitations emerge. Very low infection foci below 10 per well cause imaging algorithms to misclassify debris as real infection foci. This phenomenon raises the false-positive rate to 8–12%. Strong non-specific fluorescence derived from lipemic sera increases background noise. It further reduces the correlation between VRNT and PRNT to 0.80–0.88. Viral variants with altered cell tropism including some BA9 strains generate fewer adherent infected cells. VRNT titers accordingly underestimate PRNT results by 0.5–1 log_2_ in 20–30% of variant serum samples.

#### 2.2.2. Focus Reduction Neutralization Test (FRNT)

FRNT is an optimized neutralizing antibody detection technology based on PRNT, which replaces traditional plaques with fluorescence/chromogenic foci formed by virus-specific antigens expressed after viral infection of cells to achieve rapid quantification of neutralizing activity [17]. It is currently a mainstream method for HRSV-neutralizing antibody detection that balances accuracy and detection efficiency, and it has been included by the WHO as one of the recommended detection methods for HRSV vaccine clinical trials.

The technical principle of FRNT is consistent with that of PRNT, both of which are based on the blocking effect of antibodies on live virus infection of host cells, with the core difference being the identification and judgment method of positive signals. The standard operating procedure is as follows. First, the test serum inactivated at 56 °C is serially diluted and co-incubated with a fixed dose of HRSV 50–100 focus-forming units (FFU) in a 96-well plate at 37 °C for 1–2 h to allow sufficient combination of neutralizing antibodies in the serum with the virus; the serum-virus mixture is then inoculated onto a confluent monolayer of Vero or HEp-2 cells, and fresh cell culture medium is added after 1 h of adsorption without overlay with semi-solid agarose, followed by incubation for 24–48 h. Cells are fixed with paraformaldehyde and permeabilized, followed by incubation with specific primary antibodies against HRSV nucleoprotein (N) or fusion protein (F), and secondary antibodies are labeled with fluorescence/horseradish peroxidase for immunostaining. Finally, an automated imaging analysis system is used to count virus-infected foci in the wells, and the serum dilution that inhibits 50% of focus formation (FRNT_50_) is used as the judgment standard for neutralizing antibody titer [18].

Kim proposed crystal violet-based rapid staining combined with automated imaging analysis to address the drawbacks of prolonged HRSV infection and small plaque formation. This strategy uses high-resolution imaging and intelligent recognition algorithms to clearly distinguish HRSV-induced foci from background noise, lowering the intra-assay coefficient of variation of focus counting to below 8% and enhancing FRNT_50_ precision and repeatability [19]. Quantitatively, optimized FRNT shows intra-assay CV = 4–7%, inter-assay CV = 7–11%, and correlation with PRNT of r = 0.91–0.95 across panels of 50–200 human sera [19,20]. To further improve the assay throughput, Zielinska developed an optimized microneutralization method incorporating automated plaque counting, which replaced laborious manual counting, reduced operator bias, and improved accuracy and reproducibility. After systematic condition optimization, this assay enabled the efficient and reliable quantification of HRSV infectivity and neutralizing antibody titers, supporting large-scale serological testing and vaccine evaluation [21]. Additionally, high-throughput analytical platforms have been adopted to standardize the FRNT performance. Notably, Puglia et al. established and validated a fully automated FRNT system using the CTL Immunospot S6 Universal Analyzer for high-throughput neutralizing antibody quantification. This system delivered excellent precision, reproducibility, and specificity by standardizing the virus inoculum, incubation duration, immunostaining, and automated focus counting, providing a scalable framework for inter-laboratory harmonization. This standardized, high-throughput FRNT is readily adaptable to HRSV seroepidemiology, enabling robust, large-scale, and cross-laboratory monitoring of population antibody levels to inform public health strategies [20].

In seroepidemiological surveys, FRNT has become the preferred method for regional population HRSV-neutralizing antibody baseline surveys because of its high-throughput advantage, which can quickly clarify the distribution of immune levels in populations of different ages and regions. A clinically validated FRNT was also used to analyze cross-sectional sera collected during the 2021–2023 HRSV outbreaks, providing key seroepidemiological evidence to support the HRSV “immunity debt” hypothesis. The study demonstrated that reduced viral exposure during pandemic-related social distancing led to diminished population immunity, which contributed to the subsequent surge in HRSV epidemics, further validating the critical role of FRNT in large-scale seroepidemiological surveillance and public health decision making [22].

When FRNT yields inaccurate results, several limitations exist. A low proportion of pre-F antibodies (<30% of total anti-F antibodies) causes FRNT to overestimate neutralization by 0.5–1 log_2_ versus pre-F-specific PNT/RVS in 15–20% of post-vaccination sera. Excess non-specific IgG increases background foci, raising false-positive rates to 6–10% at dilutions < 1:40. Subtype B strains with low N-protein expression produce faint foci, leading FRNT50 to underestimate PRNT by 0.5 log_2_ in 25% of subtype B sera. Without unified standard operating procedures, inconsistent imaging thresholds cause >1 log_2_ titer discrepancies across laboratories in 30–40% of samples.

#### 2.2.3. Pseudovirus Neutralization Test (PNT)

The pseudovirus neutralization test is a live virus-free neutralization detection method developed based on viral vector engineering technology. It uses lentivirus, vesicular stomatitis virus (VSV), and other viruses as backbones and integrates the key envelope glycoproteins of HRSV (mainly F protein and some include G protein) and reporter genes to construct replication-defective HRSV pseudoviruses. Neutralizing antibody activity is quantified by detecting changes in reporter gene signals. This method avoids the biosafety risks of live viruses and has the characteristics of high throughput and easy operation, making it an important technical means for basic HRSV research and large-scale screening and is especially suitable for research institutions without BSL-2 laboratory conditions to carry out relevant detection. Multiple studies have demonstrated that pseudovirus neutralization assays show high correlation, excellent specificity, and good reproducibility with FRNT and live virus-based neutralization tests, supporting their use as safe, reliable, and high-throughput alternatives for neutralizing antibody detection [23,24,25].

Compared with traditional PRNT and FRNT, pseudovirus neutralization testing has extremely significant technical advantages, which are also the core reasons for its rapid popularization. First, it has high biosafety; pseudoviruses have no replication ability and can only complete a single round of infection. The experimental operation can be performed in BSL-1/BSL-1+ laboratories, which greatly reduces the requirements for biosafety protection and breaks the restrictions of live virus detection under experimental conditions [26,27]. Second, it has a high detection throughput and short cycle; the experimental system based on 96-well/384-well plates is compatible with fully automated liquid handling and detection platforms, which can complete the detection of more than 1000 serum samples per day, and the whole process from incubation to result takes only 1–2 days, which is far superior to traditional methods [28,29,30]. Third, it has a high specificity and can be used for targeted detection. By constructing pseudoviruses expressing only specific proteins (pre-F, G protein) or specific subtypes, this method can accurately detect neutralizing antibodies against a certain target or subtype in serum, effectively distinguish pre- and post-F conformation-specific antibodies, and provide accurate data for the evaluation of vaccine immunogenicity (antibody response induced by pre-F antigen vaccines) [31]. Fourth, it has low sample and reagent consumption; pseudoviruses have high infection efficiency, only microliter-scale serum samples are required for detection, and no expensive immunostaining reagents are needed, which greatly reduces the detection cost and is suitable for large-scale seroepidemiological surveys [32].

Quantitatively, pre-F-pseudovirus PNT correlates with PRNT with r = 0.88–0.93, intra-assay CV = 3–6%, inter-assay CV = 5–9%, with diagnostic specificity > 97% and sensitivity > 94% relative to FRNT. For pre-F-specific antibodies, PNT correlation with pre-F RVS reaches r = 0.95–0.97 [24,32].

In practical applications of HRSV-neutralizing antibody detection, pseudovirus neutralization testing has achieved multiscenario coverage and has become an important supplement to traditional live virus detection methods. In vaccine development, this method can quickly screen candidate vaccine strains and evaluate neutralizing antibody profiles induced by different antigens (pre-F, F+G fusion antigens) [33]. It can also detect neutralizing activity in large-scale samples in the early stages of vaccine clinical trials, thereby providing preliminary data for vaccine efficacy evaluation. In the development of neutralizing antibody drugs, a pseudovirus neutralization test can realize high-throughput screening of monoclonal antibody drugs and accurately detect the neutralizing activity of monoclonal antibodies against different HRSV subtypes and mutant strains, thereby providing a basis for the evaluation of broad-spectrum drug activity [34]. For example, in the detection of in vitro neutralizing activity of nirsevimab, the pseudovirus system has become a routine method to quickly verify its specific binding ability to the pre-F conformation. In seroepidemiological surveys, this method is widely used for regional population HRSV-neutralizing antibody baseline surveys because of its advantages of high throughput and low cost, which can quickly clarify the distribution of antibody levels in populations of different ages and groups, and it can be used for typing detection of A and B subtype-specific antibodies.In addition, pseudovirus neutralization testing can monitor HRSV variation. Pseudoviruses bearing F/G protein mutations from epidemic strains enable rapid assessment of population serum neutralization against variants, supporting analysis of viral pathogenicity and immune escape [35]. Accurate identification of the effects of mutations on neutralizing antibodies is critical for assessing immune evasion and guiding antibody drug optimization. A recent systematic study comprehensively defined how mutations throughout the HRSV proteome affect the activity of antibodies used for HRSV prophylaxis, enabling a more precise interpretation of neutralization test results against emerging variants [36].

Despite the prominent advantages of high safety, high throughput, and low cost, as well as its role as an important innovation in HRSV-neutralizing antibody detection that compensates for traditional live virus detection deficiencies, pseudovirus neutralization testing has technical limitations, including differences from wild virus infection characteristics, unstable F protein conformation, lack of a standardized system, and a ceiling effect in high-titer antibody detection. With the optimization of pseudovirus construction systems and establishment of standardized protocols, its detection consistency with wild viruses will improve, and it is expected to be more widely applied in HRSV-related research and detection, serving as a key complement to PRNT and FRNT.

When PNT fails or misleads, sera dominated by post-F antibodies such as those obtained in the early convalescent phase of natural infection lead pre-F-exclusive PNT to underestimate FRNT and PRNT results by 1–2 log_2_ in 30–40% of samples. Unstable pre-F pseudovirus batches experience conformational decay throughout the detection process, leading to titer reduction over 0.5 log_2_ and a decline of correlation coefficient to 0.75–0.85. High titer sera exceeding 1:2560 trigger signal saturation and the ceiling effect keeps PNT_50_ at a stable level while PRNT and FRNT values keep rising, which further underestimates the actual neutralization titer by 0.5–1 log_2_. Some subtype B sera present G protein-dependent neutralization activity and F-only PNT fails to detect such neutralizing capacity, thus underestimating FRNT outcomes by 0.5 log_2_ in 20–25% of subtype B samples.

#### 2.2.4. Fluorescent/Luminescent Reporter Virus System (RVS)

The fluorescent/luminescent reporter virus system is a detection technology that inserts fluorescent or luminescent reporter genes into non-essential regions of the wild HRSV genome using reverse genetic technology to construct recombinant live viruses that can stably express reporter proteins. The intensity of the reporter signal is used to directly reflect the level of viral infection and replication, thereby quantifying neutralizing antibody activity. This system retains the complete genomic structure and natural infection characteristics of wild HRSV and has the advantages of authentic live virus detection and visualization and quantification of reporter signals [37].

The construction of HRSV reporter viruses relies on reverse genetic manipulation technology, and its core principle is as follows. Using the full-length genomic cDNA of HRSV A/B subtype standard strains as a template, reporter genes such as green fluorescent protein (GFP) [38,39], red fluorescent protein (mCherry) [40,41,42,43], and firefly luciferase (Luc) [42] are inserted into gene spacer region [38,40,41] of the virus, and ribozyme sequences are introduced to ensure the correct cleavage and replication of the viral genome. The recombinant genomic plasmid and helper plasmids encoding HRSV structural proteins are co-transfected into susceptible cells (HEp-2, Vero cells) to complete virus rescue and amplification, and monoclonal reporter viruses are obtained by purification through the limiting dilution method. The recombinant virus can complete its entire life cycle in host cells; the expression of the reporter gene is synchronized with viral replication, and the fluorescence intensity or luminescence value of the reporter signal is in good linear positive correlation with the virus titer.

According to the different types of reporter genes, HRSV reporter virus systems are divided into main two types: fluorescent reporter viruses and luminescent reporter viruses, which are adapted to different detection scenarios. Fluorescent reporter viruses use GFP and mCherry as core reporter proteins, and the cellular localization of viral infection can be directly observed using fluorescence microscopy. Combined with a high-content imaging system, automatic counting of infection foci and quantitative analysis of fluorescence intensity can be realized, intuitively reflecting the infection range and replication level of the virus. It is suitable for visual research on antibody neutralization mechanisms, such as observing the blocking effect of neutralizing antibodies on viral adsorption, penetration, and other links. Luminescent reporter viruses use luciferase as the reporter protein, and the luminescence value is detected using a chemiluminescence instrument after adding a substrate. It has a wider dynamic range of signal detection and higher sensitivity and can realize fully automated quantification without subjective errors in manual counting. It is suitable for the detection of neutralizing antibody titers in large-scale serum samples and batch sample screening in vaccine clinical trials. At present, the most widely used are mCherry red fluorescent reporter virus and firefly luciferase reporter virus. The former has a stable fluorescent signal and low background interference, and the latter has a detection sensitivity of less than 10 PFU/mL, both of which meet the technical needs of different studies [44].

Quantitatively, luciferase RVS correlates with PRNT with r = 0.92–0.96, intra-assay CV = 3–5%, inter-assay CV = 5–8%, LOD = 1:10, and linear dynamic range = 1:10–1:5120. For pre-F-specific RVS, correlation with pre-F PNT is r = 0.95–0.98, AUC = 0.99 [44].

In the practical application of HRSV-neutralizing antibody detection, fluorescent/luminescent reporter virus systems have become a key technology connecting basic research and clinical applications, with application scenarios covering vaccine research and development, antibody drug evaluation, seroepidemiological surveys, virus variation monitoring, and other fields. In clinical trials, this system has been widely used to evaluate neutralizing antibody responses induced by vaccines. For example, in the research and development of mRNA vaccines and subunit vaccines, the neutralizing activity of vaccinated serum against reporter viruses is detected to accurately evaluate the immunogenicity and protective effect of vaccines, and typing detection of pre- and post-F-specific antibodies can be realized, providing an optimization basis for vaccine antigen design [45,46]. In the development of neutralizing antibody drugs, reporter virus systems can quickly detect the neutralizing activity of monoclonal antibody drugs against different HRSV subtypes and mutant strains and intuitively analyze the action targets and broad spectrum of drugs [8]. In seroepidemiological surveys, the luminescent reporter virus system has become one of the preferred methods for large-scale population HRSV-neutralizing antibody baseline surveys because of its advantages of high throughput and sensitivity, which can quickly clarify the distribution of antibody levels in populations of different ages, regions, and immune backgrounds and can accurately detect low-titer neutralizing antibodies, which are suitable for the evaluation of antibody levels in immunocompromised populations [47]. In virus variation monitoring, by constructing reporter viruses carrying mutation sites of F/G proteins of epidemic strains (site Ø and site II mutations of the F protein), the neutralizing activity of population serum against mutant strains can be quickly evaluated, and the immune escape trend of the virus can be determined in a timely manner, providing a scientific basis for the adjustment of HRSV prevention and control strategies [47]. In addition, this system can be used for basic research on HRSV infection mechanisms [48], such as in combination with organoid chip models to analyze the protective effect of neutralizing antibodies in the mucosal microenvironment, providing new technical means for mucosal immunity research [49,50,51,52].

When RVS fails or misleads, reporter gene attenuation occurs after more than ten passages and decreases signal intensity, which makes RVS_50_ overestimate PRNT outcomes by 0.5–1 log_2_ in 20–25% of samples. Hemolyzed sera generate high background luminescence and narrow the effective dynamic range, resulting in 8–12% of low-titer positive samples below 1:20 being wrongly judged as negative. Conformational drift of F protein in reporter virus causes the loss of pre-F epitopes and pre-F RVS underestimates authentic pre-F titers by 0.5 log_2_ in 15–20% of vaccine related sera. Aging HEp-2 and Vero cell lines undergo susceptibility changes that elevate inter-assay CV to 12–18% and lower the correlation coefficient with PRNT to 0.80–0.88.

## 3. Methodological Performance Comparison and Application Scenarios

Neutralizing antibody detection methods for HRSV differ substantially in biosafety requirements, speed, throughput, precision, and ability to detect pre-F-specific antibodies. Below, we systematically compare their core performance metrics and practical utility, then provide scenario-based selection guidance. The quantitative differences among methods reflect trade-offs between accuracy, speed, biosafety, and cost, which directly influence assay choice for vaccine trials, serosurveillance, and basic research.

### 3.1. Methodological Performance Comparison

The core performance indicators of the five mainstream HRSV-neutralizing antibody detection methods are comprehensively compared in Table 1, including detection principle, throughput, experimental cycle, biosafety level, core advantages, and main limitations. Quantitative differences in precision, correlation, and detection range among different methods reflect essential methodological differences, which directly determine their applicable research scenarios and data credibility.

### 3.2. Suggestions for Application Scenarios

Combined with the performance characteristics, biosafety level, and throughput advantages of the above detection methods, different HRSV-neutralization assays are adapted to distinct research and practical scenarios. The matching principle fully considers experimental conditions, research objectives, and detection cost, and the scenario-based method selection is summarized in Table 2.

### 3.3. Cross-Method Pre-F Centric Comparison

Currently approved HRSV vaccines and monoclonal antibodies predominantly target stabilized pre-F antigens, making pre-F-specific neutralizing antibodies the key immunological correlate of protection. It is essential to clarify differences in pre-F specificity, protective correlation, and inherent detection bias across all five mainstream assays. Table 3 summarizes these core indicators in a simplified form, while the practical implications are interpreted in the text below.

## 4. Standardization Challenges and Prospects

The rapid development of HRSV-neutralizing antibody detection technologies has provided diverse technical support for vaccine research and development, population immune monitoring, and clinical prevention and control. However, at present, the various detection methods lack a unified, standardized system. In addition, the complexity of the antigenic characteristics of the virus itself leads to poor comparability of results between different laboratories and detection platforms, which has become a core bottleneck restricting the large-scale application of HRSV-neutralizing antibody detection technologies and the intercommunication of research results. Simultaneously, with the acceleration of the clinical translation of HRSV vaccines and neutralizing antibody drugs, higher requirements have been proposed for the accuracy, comparability, and applicability of detection methods. Promoting the standardization of HRSV-neutralizing antibody detection, optimizing technical systems, and expanding application scenarios have become the focus of research and development in this field. Regarding the current status of technological development, this article analyzes the standardization challenges faced by HRSV-neutralizing antibody detection and the prospects for future technological developments and applications.

### 4.1. Limitations of Existing International Reference Materials for HRSV

WHO HRSV international standard sera (NIBSC 16/284, original; NIBSC 16/322, updated) [53] are prepared from pooled human serum donations. Donor sera are selected from healthy adults naturally infected with both HRSV-A/B, excluding acute infection and immune compromise. High-titer sera are pooled, inactivated, filtered, lyophilized, and calibrated internationally; these infection-induced pools contain mixed pre-F/post-F and non-neutralizing antibodies, representing natural immunity. Although the latest updated standard 16/322 provides assigned neutralizing antibody titers for both HRSV-A and HRSV-B subtypes to compensate for the deficiency of incomplete subtype coverage, they are prepared based on traditional laboratory strains whose antigen characteristics differ from the currently prevalent ON1 and BA9 variant strains, resulting in limited detection compatibility. Derived from pooled human polyclonal serum, this reference material lacks monoclonal antibody criteria and defined immunoglobulin subclass attributes, rendering it incapable of accurately evaluating epitope-specific antibody levels. Furthermore, it is primarily applicable for plaque reduction and microneutralization assays, with poor commutability across pseudovirus detection and immunological quantification platforms. Obtained from adult serum samples, it exhibits mismatched matrix characteristics with specimens from infants, elderly populations, and respiratory secretions, restricting its application in clinical testing. In addition, standardized international reference materials concerning intact HRSV antigens, prefusion F proteins, and nucleic acid molecules remain absent, hindering comprehensive standardization for viral identification, nucleic acid quantification, and quality evaluation of vaccines and antiviral drugs.

In-house standard sera are pooled from healthy, naturally seropositive adults, screened for high neutralizing titers against local strains, processed and stored, and assigned a relative titer for routine QC and assay normalization. There are clear differences between vaccine-induced and infection-induced anti-sera. Vaccine-induced sera, elicited mainly by prefusion F (pre-F) vaccines, are dominated by high-potency, pre-F-specific neutralizing antibodies with limited post-F and non-neutralizing reactivity. They are ideal for vaccine potency calibration, pre-F assay validation, and monitoring vaccine-elicited protective immunity. In contrast, infection-induced sera contain a mixed profile of pre-F, post-F, and non-neutralizing antibodies, reflecting natural exposure. These sera are suitable for wild-type and variant neutralization testing, population serosurveillance, and evaluating naturally acquired immunity.

### 4.2. Lack of Unified Specifications for Virus Strain Selection and Use

HRSV is divided into two antigenic subtypes, A and B, with multiple epidemic strains in each subtype. Different strains differ in their F and G protein sequences and their antigenicity. At present, there is no unified standard for the detection of viral strains in laboratories. Some use classic standard strains, such as A2 and Long, and some use local epidemic strains, leading to detection results that do not reflect the neutralizing ability of the population to the current epidemic strains. However, there is a lack of unified specifications for the preparation and calibration of viral strains for novel detection methods. For example, there are no unified requirements for the pseudovirus packaging efficiency and titer calibration method of PNT or the passage times and titer judgment standards of recombinant reporter viruses of RVS. In some laboratories, inaccurate titer calibration of viral strains leads to systematic deviations in the judgment of neutralizing antibody titers. In addition, there is a lack of unified pre-F conformation-locked virus strains for the detection of pre-F conformation-specific antibodies, and strains independently constructed by each laboratory have differences in conformational stability, which affects the accuracy and comparability of the detection results.

Strain specificity and the ability to induce broad-spectrum neutralization are critical for HRSV vaccines. Approved and advanced prefusion F-targeted vaccines consistently demonstrate superior cross-neutralizing capacity compared with earlier candidates. The bivalent pre-F vaccine Abrysvo elicits robust neutralizing responses against both subtype A and B strains, with comparable geometric mean titers against historical and contemporary isolates, supporting broad protection in older adults and maternal immunization settings. The long-acting monoclonal antibody nirsevimab targets the highly conserved pre-F site Ø and shows potent, balanced neutralization against global clinical isolates of both A and B subtypes, including BA9 variants, with minimal variation across antigenically drifted strains. In contrast, vaccines or antibodies directed against variable regions or non-stabilized F antigens often induce strain-restricted responses with limited coverage against circulating field strains. These findings highlight that antigen design focusing on stabilized, conserved pre-F epitopes is key to achieving broad-spectrum neutralization, and evaluation against multiple contemporary strains rather than a single laboratory isolate is essential to accurately reflect real-world protective breadth and guide vaccine development and immunization strategies.

### 4.3. Inconsistent Operating Procedures and Endpoint Judgment Standards of Detection Methods

The operating procedures for all types of HRSV-neutralizing antibody detection methods involve personalized adjustments between laboratories, and the setting of core parameters lacks specifications. Multiple key parameters across assays remain non-standardized, including viral inoculum dose and incubation conditions for PRNT; immunostaining, imaging and focus criteria for FRNT; pseudovirus MOI and reporter detection settings for PNT; and result interpretation criteria for RVS. Unified standard operating procedures are currently absent. The difference in endpoint judgment standards is an important reason for the deviations in the results. Some laboratories take PRNT_50_/FRNT_50_ as the core judgment index, while the endpoint judgment thresholds of novel methods are even more lacking in unified definition. For instance, laboratories adopt disparate thresholds for PNT reporter inhibition and RVS fluorescence or luminescence quantification, resulting in inconsistent titers for identical samples. Moreover, there is no unified standard for all the methods for the detection limit of low-titer antibody samples. In some laboratories, the limit of detection is set too high, leading to the misjudgment of low-titer antibody samples as negative, which affects the accuracy of the population immune baseline surveys.

### 4.4. Uneven Capabilities of Detection Personnel and Experimental Platforms

The performance of HRSV-neutralizing antibody assays is highly dependent on the technical skills of operators and configuration of experimental platforms, both of which vary widely across laboratories. Differences in personnel training background, operational proficiency, and subjective judgment in reading cytopathic effects, fluorescent foci, and reporter signals introduce unavoidable human variability. Hardware discrepancies, including differences in cell culture facilities, incubator environments, imaging systems, plate readers, and pipetting accuracy, further amplify inter-laboratory deviations. Combined with inconsistent manual operation habits and non-unified quality control measures, these differences in personnel capacity and platform conditions lead to poor reproducibility of test results, restrict the comparability of data from different studies, and hinder the standardized evaluation of vaccine immunogenicity and population immune levels.

### 4.5. Expand the Application Scenarios of Detection Technologies to Serve the Whole-Chain Prevention and Control of HRSV

In the future, HRSV-neutralizing antibody detection technologies need to overcome the limitations of single-antibody titer detection, expand application scenarios, and integrate into the whole-chain prevention and control system of HRSV. In vaccine research and development, high-precision detection technologies are used to analyze the neutralizing antibody profiles induced by vaccines, clarify the pre-F/post-F conformation specificity and subtype cross-reactivity, and provide a basis for optimizing vaccine antigen design. In the field of population immune monitoring, large-scale high-throughput detection technologies have been combined to carry out HRSV-neutralizing antibody baseline surveys in populations of different ages, regions, and immune backgrounds, draw population immune barrier maps, clarify the distribution of susceptible populations, and provide data support for the formulation of vaccination priorities. In the field of virus variation monitoring, technologies such as PNT and RVS can be used to quickly construct virus models carrying mutation sites, evaluate the neutralizing activity of population serum against mutant strains, determine the immune escape trend of the virus in a timely manner, and provide a scientific basis for the adjustment of prevention and control strategies. In the field of clinical diagnosis and treatment, individualized neutralizing antibody detection technologies have been developed to evaluate the immune status of patients and to provide a precise basis for clinical medication guidance for neutralizing antibody drugs.

## 5. Conclusions

Accurate assessment of HRSV-neutralizing antibodies is essential for vaccine evaluation, immunization strategy optimization, and population immunity surveillance. This review systematically summarizes the principles, performance, advantages, and limitations of mainstream detection methods, including PRNT, VRNT, FRNT, PNT, and RVS.

Several binding-based assays targeting pre-F and post-F antigens are available and generally easier to standardize than functional neutralization tests. These include enzyme-linked immunosorbent assays (ELISA), chemiluminescence immunoassays (CLIA), and bead-based multiplex assays using purified, conformation-stabilised pre-F and post-F proteins. They offer high throughput, low biosafety requirements, and excellent inter-laboratory reproducibility, making them suitable for large-scale serosurveillance and vaccine immunogenicity screening. However, such binding assays cannot fully replace functional neutralization tests. Their main limitations are that they measure total binding rather than neutralizing activity, so high binding titers do not always correlate with protection; they do not account for epitope accessibility or antibody avidity in a viral context; and they cannot distinguish antibodies that block viral entry from those targeting non-neutralizing epitopes. Therefore, pre-F/post-F binding assays support standardized high-throughput serology, whereas functional neutralization assays eliminate non-protective post-F antibodies to precisely evaluate authentic viral neutralization and in vivo protective potential.

## Figures and Tables

**Table 1:** Performance comparison of HRSV neutralization assays.

Detection Method	Core Principle	Throughput	Cycle	Biosafety Level	Key Strength	Key Limitation
PRNT	Live virus plaque inhibition	<100/day	5–7 d	BSL-2	Gold standard, high in vivo correlation	Slow, low throughput, subjective counting
VRNT	Automated live virus imaging	>500/day	24 h	BSL-2	Fastest live assay, high precision, strong PRNT correlation	Anti-N only, background interference
FRNT	Immunostained focus inhibition	200–500/day	2–3 d	BSL-2	Balanced speed/accuracy, WHO-recommended	High reagent cost, inter-lab variability
PNT	Replication-defective pseudovirus reporter	500–1000/day	1–2 d	BSL-1/1+	Highest biosafety/throughput, epitope-specific	No full viral cycle, conformational instability
RVS	Recombinant live reporter virus	300–500/day	2–4 d	BSL-2	Native viral cycle, high sensitivity, dynamic monitoring	High technical barrier, instrument-dependent

**Table 2:** Selection of HRSV neutralization assays for different scenarios.

Research/Application Scenario	Preferred Detection Method	Alternative Method	Selection Basis
Vaccine clinical trial efficacy evaluation/Neutralizing antibody calibration	PRNT	FRNT	Gold standard with good international comparability
Large-scale seroepidemiological survey/Population immune monitoring	FRNT	Luminescent RVS	High throughput, suitable for batch sample testing
Primary laboratory detection without BSL-2 conditions	PNT	—	Low biosafety requirement, simple and efficient
Neutralizing antibody drug development and high-throughput screening	PNT	RVS	Epitope specificity, convenient for preliminary screening
HRSV infection mechanism research	Fluorescent RVS	FRNT	Real-time dynamic observation of viral infection
Virus variation and immune escape monitoring	RVS	PNT	Capable of evaluating neutralization against variant strains
Trace sample detection	Microfluidic FRNT/RVS	PNT	Ultra-low consumption of clinical specimens

**Table 3:** Comparison of Pre-F characteristics among different assays.

Method	Pre-F Specificity	Main Detection Object	Correlation with Protection	Major Detection Bias
PRNT	No	Pre-F + post-F	Moderate	Overestimate titer by 0.5–1 log_2_
VRNT	No	Pre-F + post-F	Moderate	Background fluorescence interference
Standard FRNT	No	Pre-F + post-F	Moderate	Overestimate titer by 0.5–1 log_2_
Pre-F FRNT	Yes	Only pre-F	High	Minimal deviation
Pre-F PNT	Yes	Only pre-F	Very high	Slight underestimation
Pre-F RVS	Yes	Only pre-F	Very high	Minor underestimation

## Data Availability

No new data were created or analyzed in this study. The data supporting the findings of this study are available from the corresponding author upon reasonable request.
